# An Unusual Presentation of Cecal Volvulus With Internal Herniation Through the Foramen of Winslow

**DOI:** 10.7759/cureus.32960

**Published:** 2022-12-26

**Authors:** Arden Perabo, Andrew Alfaro, Sheldon Baty, Susan Sherali, Basem Soliman

**Affiliations:** 1 School of Medicine, Texas Tech University Health Sciences Center, Amarillo, USA; 2 Surgery, Texas Tech University Health Sciences Center, Amarillo, USA

**Keywords:** hemicolectomy, intestinal obstructions, cecal volvulus, foramen of winslow, internal hernias

## Abstract

Internal hernias through the Foramen of Winslow account for 0.1% of abdominal hernias and 8% of internal hernias, with a mortality rate of 36%-49%. Cecal volvulus accounts for only 1%-1.5% of all intestinal obstructions with a mortality rate of up to 48%. We present a case of a 56-year-old female evaluated for lower abdominal pain and nausea who received a right hemicolectomy after reduction of a cecal volvulus with internal herniation through the Foramen of Winslow. The ambiguous presentation can complicate initial management, but early detection is essential for quick operative repair and prevention of onset or progression of bowel ischemia and necrosis.

## Introduction

Internal hernias are not very common, but the high risk of strangulation and ischemia of the herniated bowel can become a surgical emergency and potentially fatal. Thus it is critical to have internal hernias higher on the differential diagnosis [1]. Internal hernias through the Foramen of Winslow account for 0.1% of abdominal hernias and 8% of internal hernias, with an associated mortality rate between 36% and 49% [2]. Two-thirds of Foramen of Winslow hernias consist of the ileum [2]. Additionally, cecal volvulus accounts for only 1%-1.5% of all intestinal obstructions [3]. Studies have demonstrated that cecal volvulus is associated with a significantly high mortality rate of up to 48% [4].

## Case presentation

**Figure 1:**
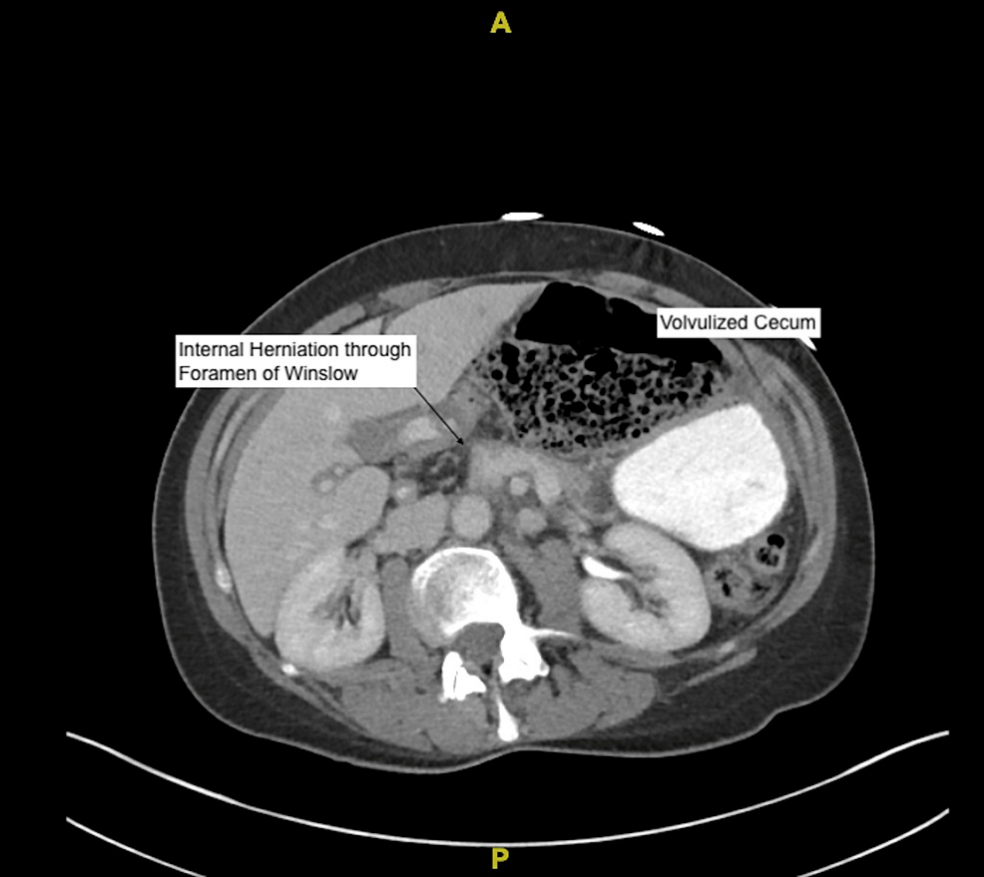
CT of the abdomen showing the cecum herniating through the Foramen of Winslow.

**Figure 2:**
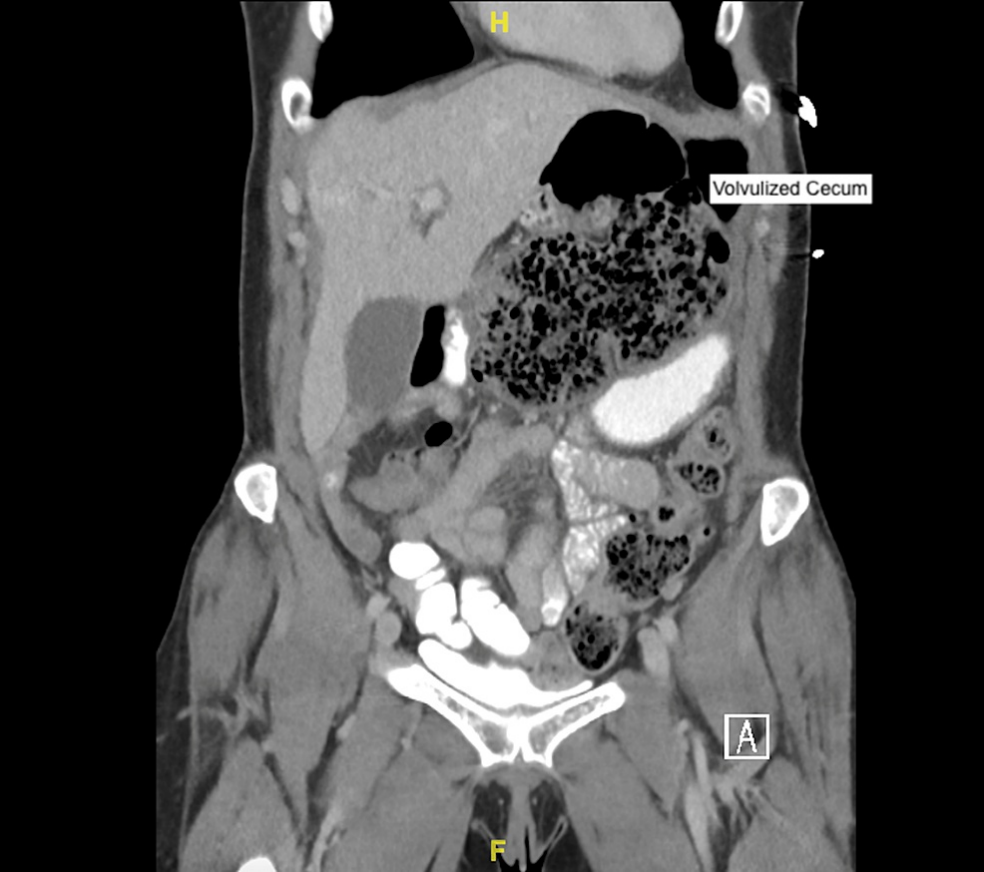
CT of the abdomen and pelvis showing cecal volvulus.

The patient is a 56-year-old female with a past medical history of anxiety and migraines. She has a past surgical history of laparoscopically assisted vaginal hysterectomy. She presented to a freestanding emergency room (ER) with five hours of constant and severe lower abdominal pain with associated nausea. She denied any previous episodes of similar symptoms. On physical exam in the freestanding ER, her abdomen was soft and nondistended with generalized tenderness. The patient received a CT of the abdomen and pelvis showing dilated small bowel loops concerning for small bowel obstruction. She was then transferred to the hospital, and a repeat CT revealed possible cecal volvulus (Figures 1-2). Upon transfer to the hospital, the patient stated that her pain progressed significantly and localized to the right lower quadrant. Physical exam at this hospital revealed right lower quadrant abdominal tenderness with rebound. 

**Figure 3:**
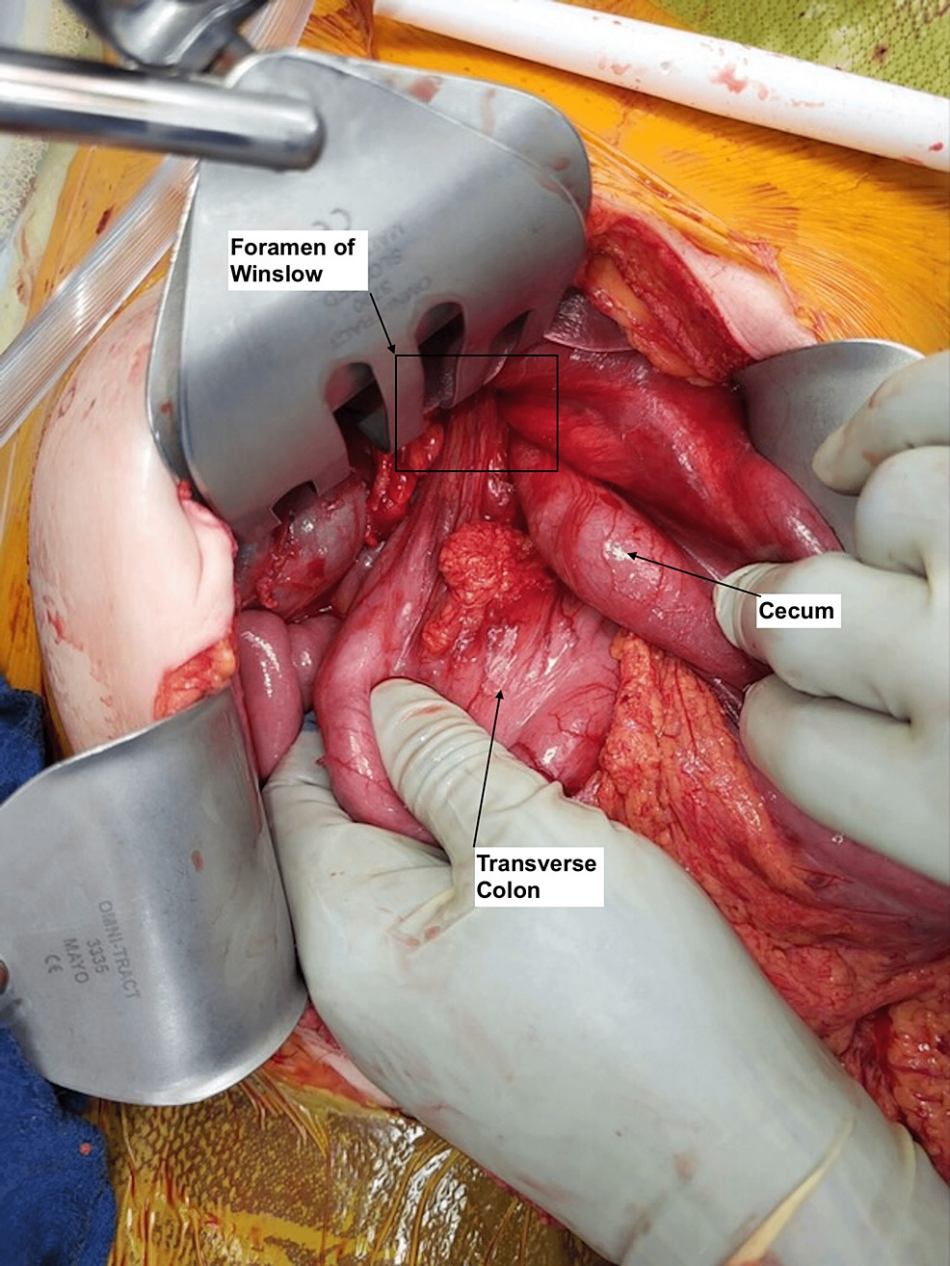
Visualization of the cecum and colon herniated through the Foramen of Winslow.

**Figure 4:**
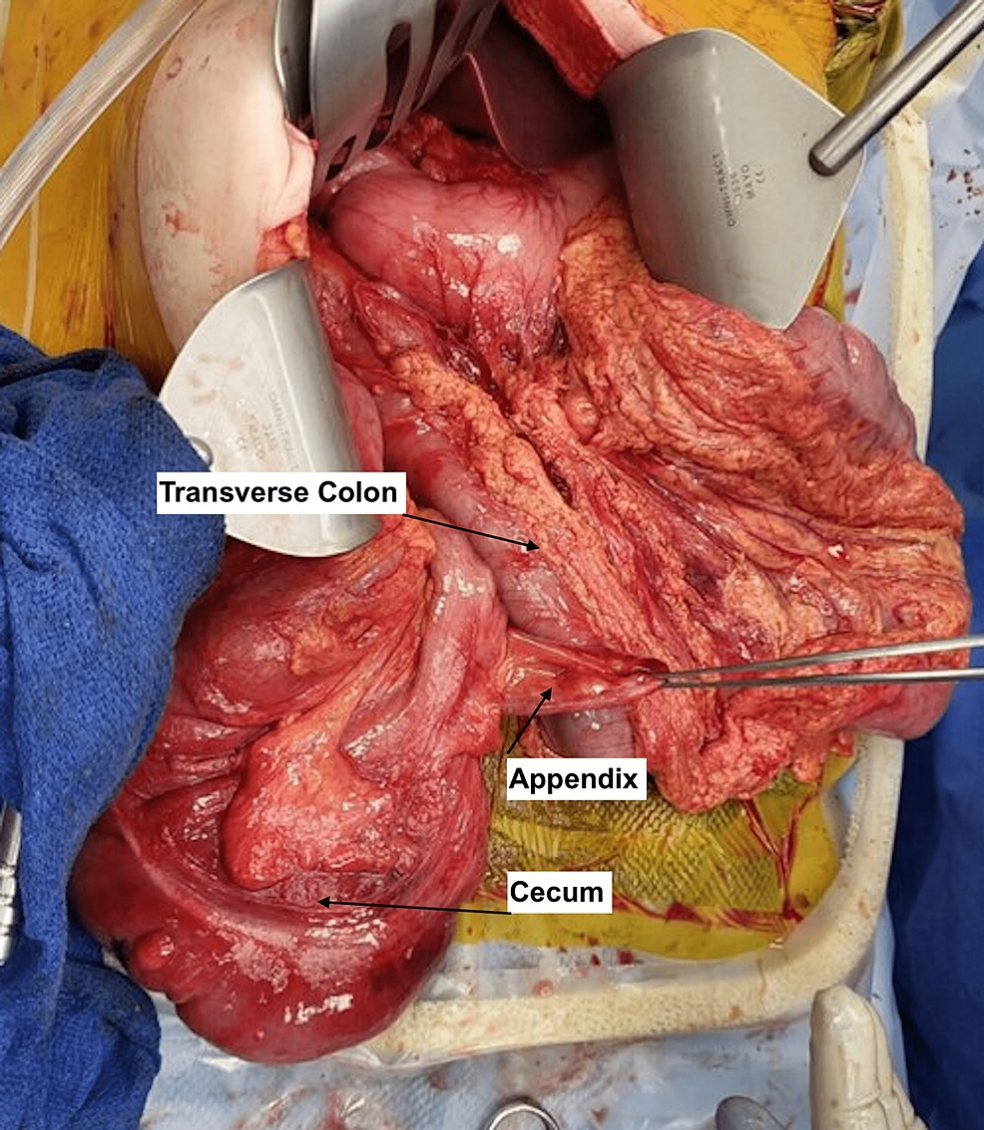
Visualization of the cecum and colon after reduction of the internal herniation.

The patient was taken to surgery for an exploratory laparotomy. During operation, it was found that the patient had a distended, dusky cecum and right colon (Figure 3), which herniated through the Foramen of Winslow. The cecum and right colon were completely reduced from the Foramen of Winslow, and attempts were made to untwist the cecum (Figure 4). However, due to the duskiness with possible early ischemia and apparent redundancy of the right and transverse colon, the decision was made to proceed with a right hemicolectomy and ileocolic anastomoses.

The patient had no further complications or significant side effects on post-op day 5 and was discharged home. Tissue pathology of the specimen resulted showing chronic inflammation and superficial mucosal ulceration consistent with clinical diagnosis of cecal volvulus. The appendix was unremarkable and there was no evidence of dysplasia or malignancy. The patient followed up in the clinic two weeks post-op. She reported improving watery stools and mild abdominal soreness but denied fever, chills, diet intolerance, nausea, or vomiting. She overall reported doing well and would return to clinic as needed.

## Discussion

Cecal volvulus is caused by twisting of the cecum, along with the terminal ileum and ascending colon, along its own axis [3]. Cecal volvulus and internal herniation through the Foramen of Winslow are both relatively rare and fatal occurrences. In this case study, we have elucidated a rare manifestation of cecal volvulus with an internal herniation through the Foramen of Winslow.

Cecal volvulus presents with relatively nonspecific signs and symptoms, as seen in this case. At the time of presentation, the patient complained of constant and severe abdominal pain and persistent nausea. There is a vast differential diagnosis for such a presentation, but rarely is cecal volvulus at the top of the list.

Risk factors for an internal herniation through the Foramen of Winslow include an abnormally long bowel, enlargement of the Foramen of Winslow, and changes in the intra-abdominal pressure [5]. The patient had an abnormally redundant right and transverse colon, and that can be the potential cause of the internal herniation. It is also possible that resection of the redundant colon, as in this case, can reduce the risk of recurrent herniation through the Foramen of Winslow in the future.

Due to their associated high mortality, cecal volvulus and internal herniation should be considered when evaluating patients with a suspected small bowel obstruction. Additionally, priority should be placed on early CT diagnosis and, if necessary, subsequent exploratory laparotomy to restore blood flow to the bowel or resect potentially ischemic portions of the gastrointestinal (GI) tract.

Through the review of literature, there are limited cases available with the unique combination of volvulus and internal herniation. In the documented cases, the ultimate management is exploratory laparotomy with reduction of volvulus from the foramen, and resection of any ischemic bowel [6]. 

## Conclusions

Internal herniation of the right colon through the Foramen of Winslow is a very rare condition with potential severe complications. These patients can present with vague gastrointestinal symptoms, such as lower abdominal pain and nausea. These nonspecific symptoms can complicate initial management, but early detection is important for quick operative repair and prevention of bowel ischemia, necrosis, and perforation.
